# Incidence of Rheumatoid Arthritis in the Southern part of Denmark from 1995 to 2001

**DOI:** 10.2174/1874312900701010018

**Published:** 2007-11-27

**Authors:** Jens K Pedersen, Anders J Svendsen, Kim Hørslev-Petersen

**Affiliations:** 1 Research Unit, King Christian X Hospital for Rheumatic Diseases, Graasten, Denmark; 2 Institute of Public Health, University of Southern Denmark, Odense, Denmark

**Keywords:** Rheumatoid arthritis, incidence, epidemiology, register

## Abstract

We estimated the incidence of rheumatoid arthritis in the southern part of Denmark from 1995 to 2001. At a rheumatology hospital serving a population of about 200 000 people over the age of 15, medical records were scrutinized. As case definition we used the tree and list format of 1987 American College of Rheumatology criteria for rheumatoid arthritis. The mean annual incidence rate per 100 000 person years was 40 in females, 21 in males, and 31 in females and males combined. The incidence of rheumatoid arthritis in Denmark is in accordance with recent studies from North America, the UK, and Northern European countries. The aetiology of rheumatoid arthritis is unknown but this study indicates that in these populations the exposure to non-genetic host and environmental aetiological factors is similar.

## INTRODUCTION

Over the last decades the incidence of rheumatoid arthritis (RA) has been studied in different populations of European origin. Using different sets of classification criteria, incidence rates per 100 000 person-years (PY) have in most studies without restrictions as to the age of the study population ranged from 26 to 58 in females, from 12 to 30 in males, and from 21 to 45 in females and males combined [1-5]. RA is seen more often in females with an overall female to male ratio of two to three. The incidence increases with age up the eighth decade and in the oldest age groups equal rates are seen in females and males [1-5, 11, 12, 14, 15].

The occurrence of RA is not static and over the past few decades studies have suggested that the incidence may be decreasing [3, 16-20]. In the light of the changing occurrence of RA, current estimates of the incidence are of general interest to doctors, epidemiologists, and health care planners.

The aim of the present study was to estimate the incidence of RA using data from a register at a rheumatology hospital in the southern part of Denmark. To get robust estimates according to age and sex, we included cases emerging over a seven-year period, from 1995 to 2001 in a population of about 200 000 people.

## MATERIALS AND METHODOLOGY

### Setting

The King Christian X Hospital for Rheumatic Diseases is a referral centre for patients with rheumatic diseases from the County of South Jutland, Denmark, a region with a population of about 200 000 people over the age of 15. To the south, the region is delineated by the Danish-German border, to the east and west by the costal line of Jutland, and to the north by two neighbouring counties.

The rheumatology hospital has for a long time been the regional centre for rheumatic expertise and a catalyst of shared care for patients with RA [21]. As a consequence, rheumatologists at the hospital have been working closely together with other health care facilities and general practitioners in the region.

In 1998 an early arthritis clinic (EAC) was introduced at the hospital. Patients aged 18 to 71 with joint swelling of at least one joint of duration of more than six weeks and less than 12 months could be referred to the clinic [22].

According to the Danish legislation, hospitals are required to keep medical records for ten years after the latest discharge or the death of a patient. At the rheumatology hospital records have been upheld irrespective of the time from the latest discharge.

From 1995 to 2001, one part-time private practising rheumatologist worked outside the hospital. From the public Health Insurance we know that this specialist treated a total of seven patients with disease-modifying anti-rheumatic drugs from 1997 to 2001. None of these patients had ever been to the rheumatology hospital and we do not know their exact diagnoses. In 2001, another rheumatologist settled in the region and we know that he diagnosed eight patients with incident RA that year. We therefore expected that the hospital register would encompass almost every new case of RA appearing in the region in the study period.

### Ascertainment of Cases

We used the list and tree format of the 1987 ACR classification criteria [23] as case definition. One of the authors (JKP) scrutinized the medical records in the hospital register for in- and outpatients with a main or secondary diagnosis of RA from 1995 to 2002. In the register the patients were registered according to the tenth revision of the International Classification of Diseases (ICD-10). The year 2002 was included to increase the chance that patients with incident RA from 2001 appeared in the register. Patients were excluded if they had been classified as having RA outside the study period, if they were under the age of 15 at the time of classification, or if they were not residing in the county.

In order to identify RA patients misclassified with other diagnoses, we also went through records for patients who, from 1995 to 2001, had been registered with a main diagnosis of polyarthrosis, other arthritis, psoriatic and enteropathic arthropathies, ankylosing spondylitis, and palindromic rheumatism.

The fulfilment of the 1987 ACR criteria was registered cumulatively and a patient was counted as an incident case the year the criteria set was fulfilled. Patients who fulfilled the criteria, but in whom it was not possible to verify the presence of joint swelling for at least six weeks due to drug treatment, were also included as cases. To ensure that the cases had RA of duration of at least six weeks, we calculated the time interval from the first symptom to classification.

The criterion of morning stiffness was registered as being fulfilled if a patient had ever had morning stiffness of at least one hour duration.

From the late 1980ies, all tests for rheumatoid factors of IgM-subtype (RF) in the region have been analyzed in the same laboratory. Throughout the period an *enzyme-linked immunosorbent assay* (ELISA) and an international reference sample had been used [24]. For the present study, we selected a cut-off at 8 IE/ml, which corresponds to the 95% percentile in normal individuals [25].

### Statistics

Incidence rates (crude rates) were calculated with the by year population in the county over the age of 15 in the denominator. Population data were provided by Statistics Denmark. The mean and annual incidence rates were reported with 95% confidence intervals using the binomial distribution. Statistics were done using Stata, version 8.2. With the *ptrend* command in Stata, we performed a test for linear trend in the annual number of cases over the study period. A two-tailed p-value of less than 5% was considered as relevant. Using the direct method, rates were standardized for age and sex to the 2002 Danish population.

### Ethical Approval

The study was approved by the local ethics committee (Reference No. 2426-02) and the Danish Data Protection Agency (Reference No. 2002-41-2231).

## RESULTS

A total of 440 cases were identified in the register and 25 (6%) had initially been registered with another diagnosis than RA (Table **1**).

Out of the 440 cases, 363 (83%) fulfilled the list and tree format of the 1987 ACR criteria, 47 (11%) fulfilled only the tree format, and 30 (7%) only the list format. In the study period, 19 additional patients were diagnosed as having RA, but they did not fulfil the classification criteria.

From 1995 to 2001, the mean annual number of cases was 63 (range: 45 to 78) in female and males combined, 21 (range: 16 to 30) in males, and 42 (range: 29 to 48) in females. After having adjusted for age, we found a statistically significant increase over the study period in the annual number of cases in females (χ²=6.53; p=0.011) and in males (χ²= 4.35; p=0.037).

In 404/440 cases (92%) it was possible to estimate the time from the first symptom associated with RA to classification. In these patients the 1% percentile for the duration of symptoms was 48 days. By year, the characteristics of the patients ascertained as cases varied but there were no systematic changes over the study period (Table **2**).

The annual incidence rates adjusted for age and sex are presented in Fig. (**1**).

Overall, the incidence rate was 40/100 000 PY in females, 21/100 000 PY in males, and 31/100 000 PY in females and males combined (Table **3**).

RA was relatively rare before the age of 35 and the rate increased up to the age group 65-74 years. RA was seen more often in females, but in the age group 75-84 years almost equal rates were observed in males and females.

## DISCUSSION

We have presented the first estimates of the incidence of RA according to age and sex in a Danish population. Although it is conceivable that almost every incident case had been ascertained at the hospital, there is an inherent risk that some patients may have been treated in general practice solely or by doctors outside the county. However, in the Danish health care system every citizen is entitled to specialist care free of charge and data from the public Health Insurance suggested that almost every RA patient in the region had been ascertained at the hospital. Consequently, we do not think that the number of patients treated by doctors outside the hospital could have been high.

The present study was retrospective and when using classification criteria the ascertainment of cases depends on the quality of data in the medical records. At the hospital 19 patients were diagnosed with RA primarily by rheumatologists who often did not make detailed notes. If they had, some of the diagnosed patients might have been discovered to have fulfilled the 1987 ACR criteria.

Central characteristics of the cases in our study did not change over the study period and this indicates that the diagnostic threshold among the doctors at the hospital did not change either. However, the possibility that we have included patients with undifferentiated arthritis as cases needs to be addressed.

In our study we included as cases some patients in whom the time criterion related to morning stiffness and joint swelling was not formally fulfilled. This approach is in line with a study from the UK where the patients were classified as having RA after one examination only [7]. The patients included in our study had symptoms associated with RA for at least six weeks prior to being classified as having RA. It is conceivable that these patients had also joint swellings in that period and, consequently, we do not think that this approach lead to the inclusion of patients with undifferentiated arthritis.

Moreover,* Wolfe, et al* studied 638 patients with undifferentiated arthritis and 503 patients with RA. In the two groups 12% and 81% were RF positive, respectively. After two years of follow-up, the disease had resolved in 54% in the group with undifferentiated arthritis and in the RA group 8% were in remission [26]. In the present study, the proportion of RF positive patients was 76%. We would therefore expect our patients to have a prognosis similar to that which was described for the RA patients in the study by *Wolfe, et al *[26].

At our hospital, it has previously been documented that the majority of the patients referred to the EAC did not have joint swellings and only 7% of the patients had undifferentiated arthritis [22]. The fraction of patients with undifferentiated arthritis was lower than what has been reported from two other EACs [27, 28]. It therefore seems unlikely that the introduction of the EAC in the study period may have lead to the incorrect inclusion of a substantial number of patients with undifferentiated arthritis in our study.

The increasing trend observed in our study may have been an artefact caused by changes in referral patterns or induced by the relatively short study period.

The EAC introduced at our hospital in 1998 received patients with recent onset arthritis. The clinic may have increased the probability that patients with RA were ascertained at the hospital. Moreover, in a study of RA patients from the UK, a decrease in the time from the first symptom to hospital referral has been described [29]. The UK study indicates a growing awareness among general practitioners of the importance of early diagnosis and treatment of RA. In our study, both the introduction of the EAC and changing medical practice among general practitioners could explain the increasing trend in the number of cases ascertained over the study period.

On the other hand, in a study from Rochester, Minnesota, a cyclic pattern in the annual incidence rates was observed from 1955 to 1995. Irrespective of the fact that overall the incidence of RA decreased over the period, time intervals of different length with either increasing or decreasing rates were observed [3]. It could be that our data, which were collected over a relatively short period of time, reflected a time interval with increasing rates in an overall pattern of decreasing rates.

The mean annual incidence described in the present study was slightly higher than what has previously been reported in studies from other Scandinavian countries, the UK, and North America, using the 1987 ACR criteria. In the following section the incidence refers to rates per 100 000 PY.

In a study from the UK using prospective notification of patients with inflammatory polyarthritis, the incidence of RA was 36 in females and 14 in males. The estimates were based on one examination only, but if the results of another examination 12 months later had also been included, the incidence was higher [7].

In Oslo, Norway, cases were identified retrospectively from a hospital register. The mean annual incidence from 1988 to 1993 was 37 in females and 14 in males [12]. In another hospital study from Northern Norway, the incidence was 36 in females and 21 in males [11]. In the Norwegian studies, it was not described whether a systematic search for misclassified cases had been performed in the registers. In other studies where register data have been used, up to 11% of the RA cases had been misclassified [1, 2, 30].

In a study from Rochester, Minnesota, the incidence was 33 in females and 26 in males from 1985 to 1995 [3]. In that setting, RA cases were identified using data from hospital records, nursing homes and private practising physicians serving the population. A similar approach was used in a study from Finland, where the incidence was 46 in females and 25 in males [8]. On the basis of data from a drug reimbursement register in Finland, the incidence of RA was 43 in females and 24 in males in 1995 [9]. In our study, the rates were close to the ones reported from Finland where data were collected from several sources including primary health care. In our opinion, this makes it plausible that almost every patient with incident RA were included in the present study.

In Denmark, the occurrence of RA has previously been investigated in a study from general practice from 1983. Using the 1958 American Rheumatism Association criteria for definite RA, the number of new patients with RA over a 13-week period was divided by the number of patients who attended the participating practices [31]. The reported proportion of patients with incident RA was 24/100 000 persons, but this measure is difficult to interpret and compare with the results of our study. It is therefore not possible to discern whether the incidence of RA in Denmark has changed over the last decades.

In our data, we observed a decrease in the rates for both male and females older than 74 years. A decrease in rates in the oldest age groups has been observed in different studies [3, 7, 11, 14] and, as previously noted by *Symmons, et al* [7], the reason for this could be that RA in older females is under-diagnosed or taken for osteoarthritis.

## CONCLUSIONS

In this study, we estimated the incidence of RA over a seven-year period around the turn of the century. We observed that the annual incidence increased over the study period. One way to evaluate if the incidence of RA is actually increasing in this population could be to prolong the observation period, using the same approach. In 2007, however, a governmental reform will unite the county with other regions and this may have influence on existing referral patterns and the definition of the population at risk. Further observation beyond 2007 may therefore be erroneous.

The mean annual incidence rates and the age- and sex specific rates were close to that which has been reported in previous studies from North America, the UK, and other northern European countries.

In populations with similar genetic constitution varying incidence rates may indicate differences in exposures to non-genetic host and environmental factors. The aetiology of RA is unknown but the present study suggests that in these populations the exposure to aetiological factors is stable.

## Figures and Tables

**Fig. (1).**
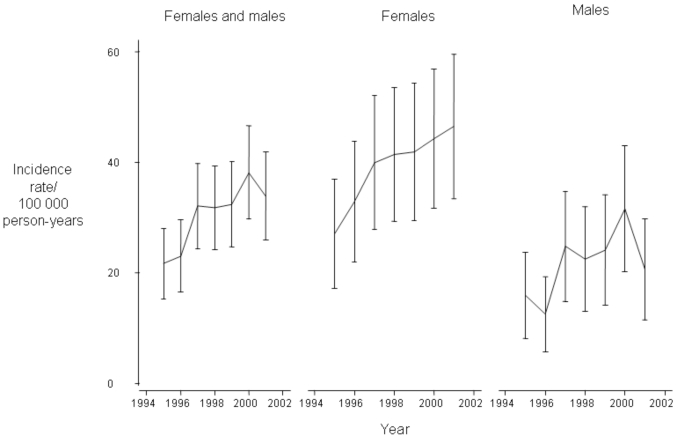
Annual Incidence Rates of Rheumatoid Arthritis from 1995 to 2001 Adjusted for Age and Sex to the 2002 Danish Population (95% Confidence Intervals by Error Bars)

**Table 1:** Incident Cases of Rheumatoid Arthritis from 1995 to 2001 According to ICD-10 Diagnoses in the Hospital Register

Disease	ICD-10 Codes	No. Cases (%)
Rheumatoid arthritis	M05.0-M05.9; M06.0-M06.9	415 (94)
Psoriatic and enteropathic arthropathy	M07.0-M07.9	4 (1)
Palindromic arthritis	M12.3	0 (0)
Other arthritis	M13.0-M13.9	21 (5)
Polyarthrosis	M15.0-M15.9	0 (0)
Ankylosing spondylitis	M45.9	0 (0)
	Total	440 (100)

**Table 2:** Characteristics in Patients with Incident Rheumatoid Arthritis from 1995 to 2001 (%, Unless Otherwise Stated)

	Year (No. cases)							
	1995 (45)	1996 (48)	1997 (66)	1998 (67)	1999 (67)	2000 (78)	2001 (79)	1995-2001 (440)
Female	64	73	63	67	66	62	71	66
Age, years, mean	60	59	62	63	61	62	60	61
Swelling ≥3 joint areas*	93	92	79	91	86	94	87	89
Symmetrical swelling*	89	94	83	91	91	96	94	91
Rheumatoid factor positive§	68	85	86	81	78	68	67	76
Rheumatic nodules*	5	4	11	9	5	6	4	7
Radiographic changes*	24	22	33	21	19	18	25	23

* According to 1987 ACR criteria (23).

§ Detected by ELISA, cut-off 8 IE/ml.

**Table 3:** Incidence Rates of Rheumatoid Arthritis According to Sex and Age in the County of South Jutland, Denmark (95% Con-fidence Intervals)

Age	No. Cases (Females/Males)	Incidence Rate/100 000 Person Years		
		Females	Males	Both
15-24	2/1	1.9 (0.2-7.0)	0.9 (0.0-5.0)	1.4 (0.3-4.1)
25-34	15/1	13.1 (7.4-21.7)	0.8 (0.0-4.5)	6.8 (3.9-11.0)
35-44	31/11	24.9 (16.9-35.3)	8.5 (4.2-15.1)	16.5 (11.9-22.3)
45-54	51/20	41.2 (30.7-54.1)	15.4 (9.4-23.8)	28.1 (21.9-35.4)
55-64	76/40	74.9 (59.0-93.8)	40.3 (28.8-54.8)	57.6 (47.6-69.1)
65-74	83/54	103.5 (82.5-128.3)	76.4 (57.6-99.5)	90.4 (75.9-106.8)
75-84	33/21	63.3 (43.6-88.9)	59.3 (36.7-90.7)	61.7 (46.3-80.3)
85+	1/0	8.6 (1.0-31.1)	0.0 (0.0-39.7)	6.2 (0.8-22.3)
15-85+	292/148	40.4 (35.9-45.3)	20.8 (17.6-24.5)	30.7 (27.9-33.7)
15-85+*		39.1 (34.7-44.0)	21.8 (18.5-25.6)	30.6 (27.8-33.6)

* Adjusted for age and sex to the 2002 Danish population.

## ABBREVIATIONS

ACR: American College of Rheumatology
EAC: Early arthritis clinic
ELISA: Enzyme-linked immunosorbent assay
ICD: International classification of Diseases
PY: Person-years
RA: Rheumatoid arthritis
RF: Rheumatoid factors
